# Microvascular Resistance Predicts Myocardial Salvage and Infarct Characteristics in ST‐Elevation Myocardial Infarction

**DOI:** 10.1161/JAHA.112.002246

**Published:** 2012-08-24

**Authors:** Alexander R. Payne, Colin Berry, Orla Doolin, Margaret McEntegart, Mark C. Petrie, M. Mitchell Lindsay, Stuart Hood, David Carrick, Niko Tzemos, Peter Weale, Christie McComb, John Foster, Ian Ford, Keith G. Oldroyd

**Affiliations:** From West of Scotland Heart and Lung Centre, Golden Jubilee National Hospital, Clydebank, Scotland, UK (A.R.P., C.B., M.M., M.C.P., M.M.L., S.H., D.C., N.T., C.M., J.F., K.G.O.); BHF Glasgow Cardiovascular Research Centre, Institute of Cardiovascular and Medical Sciences, Scotland, UK (A.R.P., C.B., D.C., N.T.); Robertson Centre for Biostatistics, University of Glasgow, Scotland, UK (O.D., I.F.); Siemens Healthcare Frimley Surrey, England, UK (P.W.)

**Keywords:** microcirculation, primary percutaneous coronary intervention, magnetic resonance imaging, myocardial infarction, ventricular remodeling

## Abstract

**Background:**

The pathophysiology of myocardial injury and repair in patients with ST‐elevation myocardial infarction is incompletely understood. We investigated the relationships among culprit artery microvascular resistance, myocardial salvage, and ventricular function.

**Methods and Results:**

The index of microvascular resistance (IMR) was measured by means of a pressure‐ and temperature‐sensitive coronary guidewire in 108 patients with ST‐elevation myocardial infarction (83% male) at the end of primary percutaneous coronary intervention. Paired cardiac MRI (cardiac magnetic resonance) scans were performed early (2 days; n=108) and late (3 months; n=96) after myocardial infarction. T_2_‐weighted‐ and late gadolinium–enhanced cardiac magnetic resonance delineated the ischemic area at risk and infarct size, respectively. Myocardial salvage was calculated by subtracting infarct size from area at risk. Univariable and multivariable models were constructed to determine the impact of IMR on cardiac magnetic resonance–derived surrogate outcomes. The median (interquartile range) IMR was 28 (17–42) mm Hg/s. The median (interquartile range) area at risk was 32% (24%–41%) of left ventricular mass, and the myocardial salvage index was 21% (11%–43%). IMR was a significant multivariable predictor of early myocardial salvage, with a multiplicative effect of 0.87 (95% confidence interval 0.82 to 0.92) per 20% increase in IMR; *P*<0.001. In patients with anterior myocardial infarction, IMR was a multivariable predictor of early and late myocardial salvage, with multiplicative effects of 0.82 (95% confidence interval 0.75 to 0.90; *P*<0.001) and 0.92 (95% confidence interval 0.88 to 0.96; *P*<0.001), respectively. IMR also predicted the presence and extent of microvascular obstruction and myocardial hemorrhage.

**Conclusion:**

Microvascular resistance measured during primary percutaneous coronary intervention significantly predicts myocardial salvage, infarct characteristics, and left ventricular ejection fraction in patients with ST‐elevation myocardial infarction. **(*J Am Heart Assoc*. 2012;1:e002246 doi: 10.1161/JAHA.112.002246)**

## Introduction

Salvaging threatened myocardium after acute coronary occlusion is a key therapeutic objective,^1^ and the amount of salvageable myocardium is an independent predictor of prognosis.^2^ Despite successful primary percutaneous coronary intervention (PCI), at least one third of patients subsequently develop microvascular obstruction that portends an adverse prognosis.^2,3^

Ex vivo studies in experimental animals have suggested that maintained microvascular patency in ischemic myocardium limits infarct size.^4^ However, in vivo human studies are lacking because it has not been possible to measure coronary microvascular function directly. Standard clinical methods for assessing the efficacy of reperfusion, such as the electrocardiogram^5^ and angiographic perfusion grade,^6^ are indirect and have limited diagnostic accuracy for culprit artery microvascular function. Microvascular damage revealed noninvasively by contrast‐enhanced cardiac magnetic resonance (CMR) imaging is an independent predictor of prognosis after acute myocardial infarction (MI).^3^ However, because of issues around feasibility and cost, CMR is not normally performed early after MI. When CMR is available, it is usually performed up to 7 days after hospital admission in medically stabilized patients. Therefore, a direct quantitative measure of microvascular function during primary PCI would be an important advance for very early risk stratification in routine clinical practice.

The index of microvascular resistance (IMR) is a simple coronary guidewire–based method for assessing coronary microvascular function,^7–11^ including in patients with ST‐elevation MI (STEMI).^11^ IMR measured at the end of primary PCI is an independent predictor of left ventricular ejection fraction as measured by echocardiography^10^ or CMR^11^ 3 months after PCI. These observations suggest that IMR can be used to study the pathophysiology of microvascular function in STEMI and that IMR could have utility for very early prognostication.

Given that myocardial salvage can be measured with T_2_‐weighted CMR,^2,12–16^ we aimed to investigate the ability of acutely measured IMR to predict subsequent myocardial salvage and left ventricular remodeling in a cohort of patients with STEMI. Our first hypothesis was that microvascular resistance measured by IMR after culprit artery reperfusion is a determinant of subsequent myocardial salvage. Our second hypothesis was that IMR is a predictor of left ventricular function during follow‐up. Our final hypothesis was that IMR would predict the occurrence and severity of infarct characteristics, such as myocardial hemorrhage and microvascular obstruction, which have prognostic importance.^2,3^

## Methods

### Patient Population and STEMI Management

Consecutive acute STEMI patients undergoing primary PCI at a regional cardiac center were screened. The inclusion criteria were an indication for primary PCI for acute STEMI due to a history of symptoms consistent with acute myocardial ischemia and with supporting changes on the electrocardiogram (ie, ST‐segment elevation or new left bundle‐branch block).^17^ Exclusion criteria represented standard contraindications to CMR, including an estimated glomerular filtration rate <30 mL/min per 1.73 m^2^. The project was approved by the West of Scotland Research Ethics Committee.

Acute STEMI management followed contemporary guidelines.^17^ Aspiration thrombectomy, direct stenting, antithrombotic drugs, and other therapies were administered according to clinical judgment.

### IMR Measurement After Coronary Reperfusion

A coronary pressure‐ and temperature‐sensitive guidewire (St Jude Medical, Uppsala, Sweden) was used as the primary guidewire. The guidewire was calibrated outside the body, equalized with aortic pressure at the ostium of the guide catheter, and then advanced to the distal third of the culprit artery. IMR is defined as the distal coronary pressure multiplied by the mean transit time of a 3‐mL bolus of saline at room temperature during maximal coronary hyperemia, measured simultaneously (mm Hg · s, or units).^7–11^ Hyperemia was induced by 140 μ/kg per minute of intravenous adenosine preceded by a 2‐mL intracoronary bolus of 200 μg nitrate. The mean aortic and distal coronary pressures were recorded during maximal hyperemia. Previous studies in patients with stable coronary disease have established that IMR is repeatable and independent of hemodynamic variations, including heart rate, blood pressure, and myocardial contractility.^8,9^ In our study, the repeatability of IMR was assessed by duplicate measurements 5 minutes apart in a subset of 12 consecutive patients.

### CMR Acquisition and Analyses

CMR was performed approximately 2 days after MI and 3 months after MI on a Siemens MAGNETOM Avanto (Erlangen, Germany) 1.5‐Tesla scanner with a 12‐element phased‐array cardiac surface coil. The imaging protocol included cine MRI with steady‐state free precession, edema imaging with bright‐blood T_2_‐weighted CMR,^12,18,19^ and delayed‐enhancement phase‐sensitive inversion‐recovery pulse sequences.^20^ The bright‐blood T_2_‐weighted ACUT2E method (Acquisition for Cardiac Unified T_2_ Edema)^18,19^ incorporates elements of the bright‐blood^2007^ and turbo spin‐echo methods.^22^ Typical imaging parameters were as follows: acquisition time 7 to 12 seconds, matrix 192×192, flip angle 180°, echo time 1.69 ms, and bandwidth 789 Hz/pixel. Twenty‐nine coherent spin echoes (echo train length) were obtained per heartbeat, and the time interval (echo spacing) between the 180° inversion pulses was 3.4 ms. Data were acquired every second RR interval. The voxel size was 1.9×1.9×6 mm^3^. Microvascular obstruction was defined as a central dark zone on early delayed‐enhancement imaging 1, 3, 5, and 7 minutes after contrast injection and within an area of late gadolinium enhancement (LGE). MI was imaged by using a segmented phase‐sensitive inversion‐recovery turbo fast low‐angle shot^18^ radiofrequency pulse sequence 15 minutes after intravenous injection of 0.10 mmol/kg gadoterate meglumine (Gd^2+^‐DOTA, Dotarem, Guebert S.A.). Typical imaging parameters were as follows: matrix 192×256, flip angle 25°, echo time 3.36 ms, bandwidth 130 Hz/pixel, echo spacing 8.7 ms, and trigger pulse 2. The voxel size was 1.8×1.3×8 mm^3^.

### MR Image Analyses

The images were analyzed on a Siemens workstation by 2 cardiologists (A.R.P., C.B.) with 3 and 5 years’ CMR experience, respectively.^12,19,23^ These cardiologists have considerable CMR experience in patients with acute MI, including protocols with bright‐blood T_2_‐weighted CMR. A third CMR trained‐cardiologist (N.T.) provided an independent opinion when disagreement occurred, and agreement was established by consensus. All of the cardiologists were blinded to the PCI and IMR results. Left ventricular dimensions, volumes, and ejection fractions were quantified with computer‐assisted planimetry.

Reference ranges used in the laboratory were 105 to 215 g for left ventricular mass in men, 70 to 170 g for left ventricular mass in women, 77 to 195 mL for left ventricular end‐diastolic volume in men, 52 to 141 mL for left ventricular end‐diastolic volume in women, 19 to 72 mL for left ventricular end‐systolic volume in men, and 13 to 51 mL for left ventricular end‐systolic volume in women.

### Myocardial Edema and Standardized Measurements of T_2_‐Weighted Area at Risk

Quantitative assessments were performed by 2 independent observers, and their results were averaged. The approach to image analysis, including interobserver agreement, has been reported previously.^12,19^ The jeopardized area at risk on each axial image was defined as the percentage of left ventricular area delineated by the hyperintense zone on T_2_‐weighted images.^12,19^

### Infarct Definition and Size

The presence of acute infarction was established with CMR on the basis of abnormalities in cine wall motion, rest first‐pass myocardial perfusion, and LGE imaging. In addition, supporting changes on the electrocardiogram and coronary angiogram were required. Acute infarction was considered present only if LGE was confirmed on both the short‐ and long‐axis acquisitions. The myocardial mass of LGE (in grams) was quantified by a semiautomatic detection method that used a signal intensity threshold of >2 standard deviations (SDs) above a remote reference region.^11,15,19^ Infarct regions with evidence of microvascular obstruction were included within the infarct area. Microvascular obstruction was classified as relevant (central dark zone with a subendocardial or intramural distribution) or nonrelevant (dots or nil) and also was expressed as a percentage of total left ventricular mass.

### Myocardial Salvage

Myocardial salvage was calculated by subtraction of percent infarct size from percent area at risk.^12^ The myocardial salvage index was calculated by dividing the myocardial salvage area by the initial area at risk.

### Definition and Detection of Hemorrhage on T2‐Weighted CMR

Myocardial hemorrhage revealed by bright‐blood T_2_‐weighted CMR was defined as a confluent dark zone with a mean signal intensity <2 SDs of the mean signal intensity of the surrounding affected brighter area.^23^ Hemorrhage was expressed as a percentage of total left ventricular mass.

### Angiographic Analyses

All angiograms were analyzed by 2 interventional cardiologists independent of the CMR analyses. The Thrombolysis In Myocardial Infarction (TIMI) flow classification^24^ and corrected TIMI frame count^25^ were used to grade culprit artery flow at initial angiography and at the end of the PCI. The TIMI myocardial perfusion grade was used to assess myocardial perfusion.^26^

### Statistical Analyses

The primary end point was myocardial salvage index. The sample size estimate was based on the adjusted effect (multiple regression) for the relationship between IMR measured acutely and myocardial salvage index as revealed by contrast‐enhanced CMR, after adjustment for prespecified known prognostic clinical confounders. In multiple‐regression models for myocardial salvage index, 80 subjects would provide ≥80% power to detect a correlation of 0.3 with a type 1 error rate of 0.05. With allowance for occasional problems with data acquisition, the projected sample size was ≥100 subjects. All subjects with available data were used in models fitted for CMR parameters at initial and follow‐up scans. Normality was assessed by using the Shapiro‐Wilk test. Means and SDs were used to summarize normally distributed data, geometric means and SDs to describe skewed data, percentages to summarize distributions, and Pearson coefficients (*r*) to describe correlations between log IMR and CMR parameters. Normal distributions were achieved where necessary by logarithmic transformation. All tests were 2 tailed, with a significance level of 0.05.

Between‐group comparisons of continuous variables used Student *t* tests or Mann‐Whitney tests, whereas differences in proportions were assessed with either a Fisher exact test or a χ^2^ test with continuity correction, as appropriate. Random‐effects models were used to compute interrater reliability measures (intraclass correlation coefficient [ICC]) for CMR parameters measured independently by 2 observers in 20 randomly selected patients. Univariable and multivariable regression models were constructed to determine the prognostic significance of IMR with CMR parameters: myocardial salvage index, infarct size, microvascular obstruction, hemorrhage, area at risk, and left ventricular ejection fraction and volumes. Further subgroup analysis explored patients with and without a culprit left anterior descending coronary artery at initial angiography and patients with and without an occluded culprit coronary artery. We used a backward variable selection process with the cut point of 0.1 to select variables for the final multivariable models. IMR was specified in each multivariable model, but because renal function and serum troponin are not available at the time of primary PCI when IMR is measured, these variables were not included. Multivariable models adjusted for variables in stepwise selection models. For all baseline CMR outcomes, adjustment was made for the following variables: previous MI, sex, pain‐to‐balloon time, door‐to‐balloon time, thrombectomy, catheterization laboratory pulse pressure, TIMI flow, corrected TIMI frame count, and percent of ST elevation still present after PCI. For all follow‐up CMR outcomes, we adjusted for baseline value of the CMR parameter, age, door‐to‐balloon time, glycoprotein IIb/IIIa, TIMI flow, and percent of ST elevation still present after PCI.

IMR associations were described by an effect estimate (either additive or multiplicative) on the continuous CMR parameters per 20% increase in IMR, with 95% confidence intervals (CIs) and *P* values. All statistical analyses were performed in SAS (SAS Institute, Cary, NC).

## Results

### Patient Characteristics

One hundred eight patients with acute STEMI treated by primary PCI were enrolled between October 1, 2009, and July 8, 2010 (Table 1). Forty‐seven patients (44%) had anterior STEMI, and 61 patients (56%) had nonanterior MI (*P*=0.1). IMR was measured at the end of primary PCI in all patients. Intravenous adenosine was well tolerated in all patients, and there were no adverse events. All patients were alive after 3 months’ follow‐up.

**Table 1.** Baseline Characteristics of Patients With Acute STEMI and a CMR Scan at Baseline (n=108)

Age, y	57.8±10.2
Body mass index, kg/m^2^*	26 (23–30)
Heart rate, bpm	76±16
Systolic blood pressure, mm Hg*	131 (113–149)
Diastolic blood pressure, mm Hg*	80 (66–91)
Pain‐to‐balloon time, min*	186 (137–331)
IMR, mm Hg/s*	26 (17–41)
Sex, male	90 (83.3)
Previous MI	4 (3.7)
Diabetes mellitus	5 (4.6)
Sustained ventricular arrhythmias or clinical signs of acute left ventricular failure	18 (16.7)
Culprit artery	
Left anterior descending	47 (43.5)
Circumflex	13 (12)
Right coronary artery	48 (44.4)
Killip class	
I	96 (88.8)
II	2 (1.9)
III	9 (8.3)
IV	1 (1)
TIMI grade before PCI	
0/1	70 (65)
2	22 (20)
3	16 (15)
TIMI grade after PCI	
0/1	1 (1)
2	9 (8)
3	98 (91)

Data are given as mean±SD, n (%), or

* median (interquartile range).

CMR was performed during the index hospitalization (n=108) and 3 months afterward (n=96). The clinical characteristics of the patients with paired CMR scans at baseline and at follow‐up (n=96) were similar to those of patients with a single scan at baseline (n=108).

### IMR Immediately After Primary PCI

Repeated IMR measurements obtained by 4 different operators in 12 STEMI patients were highly correlated (*r*=0.99, *P*<0.001), with a mean difference between IMR measurements of 0.01 (mean standard error 1.59 [95% CI −3.52 to 3.54], *P*=0.48). The median (interquartile range) IMR in all patients was 28 (17–42) mm Hg/s, with a range of 9 to 149 mm Hg/s. IMR correlated with time from symptom onset to coronary reperfusion (*r*=0.22, *P*=0.021). The median (interquartile range) arterial pressure measured simultaneously with IMR was 79.5 (69.5–97.0) mm Hg. Arterial pressure and IMR were not correlated (*r*=0.17, *P*=0.10).

**Figure 1.**
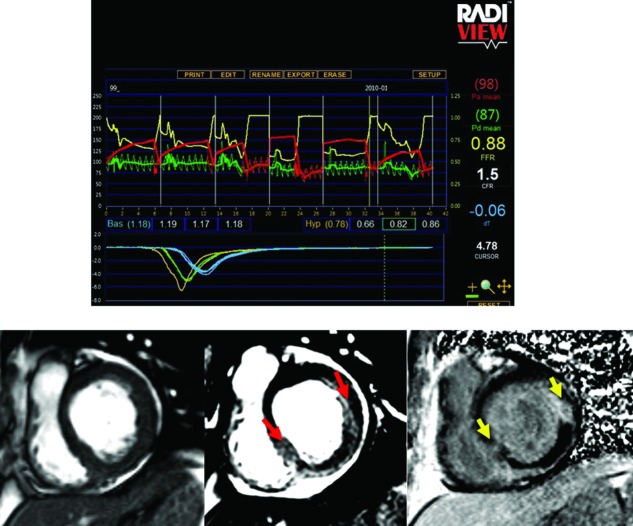
A 60‐year‐old man presented with an acute infero‐lateral STEMI. Primary PCI to the occluded left circumflex artery included aspiration thrombectomy, intravenous tirofiban, and implantation of a bare metal stent. The initial 0.014‐inch coronary guidewire was exchanged for a pressure wire (Certus, St Jude Medical, Uppsala, Sweden), and intravenous adenosine (140 μg/kg per minute) was administered. On the RADIView recording (top), Pa (red) is the aortic pressure measured from the guide catheter; Pd (green), the distal coronary pressure measured from the distal end of the pressure wire; and FFR (yellow), the fractional flow reserve derived from Pa divided by Pd. These parameters are depicted in the upper graph (Y‐axis left is pressure [mm Hg]; Y‐axis right is units). The lower graph displays coronary thermodilution curves measured with the microelectrode at the distal end of the pressure‐ and temperature‐sensitive Certus wire. The thermodilution curves are blue under resting conditions and yellow during hyperemia. The distal coronary pressure was 87 mm Hg, the mean hyperemic transit time was 0.78 seconds, and the IMR was 68 mm Hg seconds, which is markedly elevated. Two days later, CMR was performed. Cine MRI (lower left) revealed an infero‐lateral left ventricular wall motion abnormality. Matched diastolic phase images obtained by using bright‐blood T_2_‐weighted CMR (lower middle, red arrows) and LGE imaging (lower right, yellow arrows) revealed transmural edema and infarction, respectively. The area at risk revealed by bright‐blood T_2_‐weighted CMR (lower middle) was 58% of left ventricular mass. Infarct size revealed by LGE (lower right) was 57%, consistent with 1% of salvageable myocardium. The central dark zone within the infarct territory represents extensive confluent microvascular obstruction complicated by myocardial hemorrhage, indicative of severe MI. The microvascular obstruction was confirmed in orthogonal long‐axis views (not shown). The initial left ventricular ejection fraction and normalized end‐systolic volume were 49% and 39 mL/m^2^, respectively. Three months later, the left ventricular ejection fraction remained reduced (51%), and the normalized left ventricular end‐systolic volume had increased to 44 mL/m^2^, consistent with adverse left ventricular remodeling.

The Figure 1 illustrates observations in a patient with infero‐lateral STEMI, including the IMR value measured at the end of primary PCI and subsequent myocardial salvage by CMR 2 days later.

### CMR Findings

One hundred eight patients underwent CMR an average of 2 days after primary PCI. Ninety‐six patients (89%) returned for a follow‐up scan ≍3 months later (Table 2). The baseline characteristics of the patients who did or did not attend the 3‐month follow‐up MRI scan were similar. In an assessment of interobserver agreement, 20 patients were randomly selected for independent analysis by 2 observers. ICCs indicated a high degree of reliability: left ventricular ejection fraction, ICC=0.97; left ventricular end‐diastolic volume, ICC=0.98; left ventricular end‐systolic volume, ICC=0.98; and infarct size, ICC=0.99. The median (interquartile range) initial area at risk estimated by bright‐blood T_2_‐weighted CMR was 32% (24%–41%) of left ventricular mass, and the myocardial salvage index was 21% (11%–43%) (Table 2). The area at risk was greater in patients with anterior MI than in patients with nonanterior MI (mean difference 11.1% of left ventricular mass [95% CI 6.7 to 15.5], *P*<0.001).

**Table 2.** CMR Findings in 96 Patients With a Paired CMR Scan at Baseline and at Follow‐Up

MRI findings during index hospitalization	
Area at risk, %	32.9±12.4
Infarct size, %	23.5±14.2
Myocardial salvage index, %*	19.2±36.3
Microvascular obstruction, n (%)	38 (40)
Microvascular obstruction, %*	3.1±2.9
Myocardial hemorrhage, n (%)	44 (47)
Myocardial hemorrhage, % of left ventricle*	2.5±1.9
Left ventricular mass, g	
Men	141.6±27.4
Women	101.0±23.0
Left ventricular ejection fraction, %	51.3±10.0
Left ventricular end‐diastolic volume, mL	
Men	165.6±31.8
Women	125.9±33.3
Left ventricular end‐systolic volume, mL	
Men	83.1±24.6
Women	57.1±29.1
MRI findings at 3 months	
Myocardial salvage index, %*	42.11±47.5
Infarct size, %	16.6±11.6
Left ventricular ejection fraction, %	56.9±9.9
Left ventricular end‐diastolic volume, mL	
Men	174.0±39.4
Women	129.6±37.8
Left ventricular end‐systolic volume, mL	
Men	79.5±33.6
Women	51.5±31.8

Data are given as n (%) or mean±SD.

* Values are geometric mean±SD.

The CMR findings in patients with paired scans (n=96) were similar to those of patients with a single scan at baseline (n=108).

### Microvascular Resistance and CMR Findings Early (2 Days) After MI

The patients were divided into quartiles according to IMR. With higher values of IMR, the area at risk and initial infarct size increased and left ventricular ejection fraction decreased

(Table 3). Median (interquartile range) IMR was higher in patients with myocardial hemorrhage than in those with no hemorrhage: 29.1 (18.2–46.5) versus 17.1 (13.2–23.5); *P*=0.006. IMR correlated positively with the presence of microvascular obstruction (*r*=0.38, *P*<0.001) and with myocardial hemorrhage (*r*=0.43, *P*<0.001) and negatively with myocardial salvage index (*r*=−0.38, *P*<0.001).

**Table 3.** CMR at Day 2 by Quartiles of IMR Measured at the End of Primary PCI

	Quartiles of IMR					
			
	1	2	3	4	P*	*P* Trend
	(≤17)	(18–27)	(28–42)	(>42)		
Area at risk, %	30.0±12.4	28.8±11.6	31.2±11.5	38.2±12.2	0.036†	0.020
Infarct size, %	17.3±13.1	18.1±13.0	24.5±12.0	31.7±13.0	0.001‡	<0.001
Myocardial salvage index, %*	28.3±40.2	17.5±48.2	21.3±37.6	14.3±24.8	0.338‡	<0.001
Left ventricular ejection fraction, %	53.6±8.4	54.3±10.3	51.0±9.8	46.1±9.3	0.017†	0.005

Data are mean±SD, *P* value for group differences, and *P* value for test of trend.

* Values are geometric mean±SD calculated from the log‐transformed distributions.

† *P* values from analysis of variance.

‡ *P* values from Kruskal‐Wallis tests.

### Microvascular Resistance and CMR Findings Late (3 Months) After MI

At 3 months, IMR correlated negatively with left ventricular ejection fraction (*r*=−0.27, *P*=0.009) and positively with left ventricular end‐systolic volume (*r*=0.24, *P*=0.019).

### Univariable and Multivariable Relationships Between IMR and Infarct Characteristics

IMR was a univariable predictor of area at risk and early infarct size, myocardial salvage, microvascular obstruction, hemorrhage, and left ventricular ejection fraction. IMR was a univariable predictor of infarct size and left ventricular ejection fraction at 3 months (Table 4). At 2 days, multivariable models confirmed significant predictive effects of IMR on initial injury characteristics, including area at risk, infarct size, myocardial salvage, microvascular obstruction, and myocardial hemorrhage (Table 5). At 3 months, after adjustment for values at 2 days, IMR continued to show a significant predictive effect on infarct size. In separate linear models of IMR and initial infarct size, microvascular obstruction, and hemorrhage, and after adjustment for left ventricular mass, IMR was more closely associated with infarct size (data not shown) than with either hemorrhage or microvascular obstruction (*P*<0.001 for both comparisons).

**Table 4.** Estimated Effects on CMR Parameters Associated With a 20% Increase in IMR (mm Hg/s) Derived From Univariable Models

	*r*	Effect of IMR	95% CI	*P*
Univariable associations, early CMR (2 days)				
Area at risk, %	0.29	1.07	0.39 to 1.76	0.003
Infarct size, %	0.45	1.84	1.12 to 2.56	<0.001
Myocardial salvage index, %	−0.41	−3.42	−4.93 to −1.92	<0.001
Microvascular obstruction, %	0.35	0.37	0.18 to 0.56	<0.001
Hemorrhage, %	0.43	0.28	0.17 to 0.40	<0.001
Left ventricular ejection fraction, %	−0.27	−0.80	−1.35 to −0.24	0.005
Univariable associations, late CMR (3 months)				
Infarct size, %	0.34	1.13	0.48 to 1.79	<0.001
Myocardial salvage index, %	−0.31	−2.46	−4.01 to −0.92	0.002
Left ventricular ejection fraction, %	−0.27	−0.77	−1.34 to −0.20	0.009

Results are Pearson correlation coefficients (*r*) and additive effects.

**Table 5.** Estimated Effects on CMR Parameters Associated With a 20% Increase in IMR (mm Hg/s) Derived From Multivariable Models With Adjustment for Confounders at Both Time Points

	IMR Effect	95% CI	*P*
Multivariable associations,* early CMR (2 days)			
Area at risk, %	0.98	0.33 to 1.64	0.004
Infarct size, %	1.68	1.01 to 2.34	<0.001
Myocardial salvage index, %	−3.43	−4.86 to −2.00	<0.001
Microvascular obstruction, %	0.21	0.02 to 0.40	0.028
Hemorrhage, %	0.19	0.08 to 0.31	0.002
Left ventricular ejection fraction, %	−0.64	−1.16 to −0.12	0.017
Multivariable associations,† late CMR (3 months)			
Infarct size, %	−0.08	−0.47 to 0.31	0.680
Myocardial salvage index, %	−0.19	−1.31 to 0.94	0.744
Left ventricular ejection fraction, %	−0.19	−0.56 to 0.18	0.315

Results are additive effects on the CMR parameter.

* For all baseline CMR outcomes, adjustment was made for the following variables: previous MI, sex, pain‐to‐balloon time, door‐to‐balloon time, thrombectomy, catheterization laboratory pulse pressure, TIMI flow, corrected TIMI frame count, and percent of ST elevation still present after PCI.

† For all follow‐up CMR outcomes, we adjusted for baseline value of the CMR parameter, age, door‐to‐balloon time, glycoprotein IIb/IIIa, TIMI flow, and percent of ST elevation still present after PCI.

### Patients With Anterior or Nonanterior Infarcts

In a comparison of patients with anterior and nonanterior infarctions (Table 6), IMR was a multivariable predictor of early infarct characteristics, including infarct size, myocardial salvage, microvascular obstruction, and myocardial hemorrhage, in both groups. In patients with anterior MI, at 3 months, after adjustment for initial CMR findings, IMR predicted infarct size, myocardial salvage, and left ventricular ejection fraction (Table 6).

**Table 6.** Comparison of Anterior and Nonanterior Infarctions: Estimated Effects on CMR Parameters Associated With a 20% Increase in IMR (mm Hg/s)

	Anterior STEMI (n=47)		Nonanterior STEMI (n=61)	
		
	IMR Effect (95% CI)	*P*	IMR Effect (95% CI)	*P*
Multivariable associations,* early CMR (2 days)				
Infarct size, %	2.11 (0.89 to 3.32)	0.001	1.38 (0.64 to 2.12)	<0.001
Myocardial salvage index, %	−3.97 (−6.16 to −1.78)	<0.001	−3.28 (−5.12 to −1.43)	<0.001
Microvascular obstruction, %	0.37 (0.10 to 0.64)	0.009	0.14 (−0.09 to 0.38)	0.225
Hemorrhage, %	0.26 (0.08 to 0.44)	0.006	0.18 (0.03 to 0.32)	0.019
Left ventricular ejection fraction, %	−0.76 (−1.77 to 0.26)	0.145	−0.62 (−1.10 to −0.13)	0.016
Multivariable associations,† late CMR (3 months)				
Infarct size, %	−0.08 (−0.77 to 0.61)	0.812	0.04 (−0.41 to 0.48)	0.894
Myocardial salvage index, %	−1.07 (−2.95 to 0.81)	0.266	0.10 (−1.32 to 1.52)	0.892
Left ventricular ejection fraction, %	−0.89 (−1.53 to −0.26)	0.008	0.02 (−0.40 to 0.44)	0.921

Results are additive effects on the CMR parameters.

* For all baseline CMR outcomes, adjustment was made for the following variables: previous MI, sex, pain‐to‐balloon time, door‐to‐balloon time, thrombectomy, catheterization laboratory pulse pressure, TIMI flow, corrected TIMI frame count, and percent of ST elevation still present after PCI.

† For all follow‐up CMR outcomes, we adjusted for baseline value of the CMR parameter, age, door‐to‐balloon time, glycoprotein IIb/IIIa, TIMI flow, and percent of ST elevation still present after PCI.

### Patients With Occluded or Patent Culprit Arteries at Presentation

In a comparison of patients with an occluded culprit artery (TIMI flow grade 0/1) at initial presentation to those with a patent culprit artery (TIMI flow grade 2/3) (Table 7), IMR was a multivariable predictor of early infarct characteristics, including infarct size, microvascular obstruction, and hemorrhage, in both groups. At 3 months, IMR predicted myocardial salvage in patients with an occluded artery but not after adjustment for the initial extent of salvage (data not shown).

**Table 7.** Comparison of Patients With Occluded (TIMI 0/1) and Patent (TIMI 2/3) Culprit Arteries at Presentation: Estimated Effects on CMR Parameters Associated With a 20% Increase in IMR (mm Hg/s)

	Patients With an Occluded Culprit Artery(n=70)		Patients With a Patent Culprit Artery(n=38)	
		
	IMR Effect (95% CI)	*P*	IMR Effect (95% CI)	*P*
Multivariable associations,* early CMR (2 days)				
Infarct size, %	1.56 (0.69 to 2.43)	<0.001	1.97 (0.94 to 2.99)	<0.001
Myocardial salvage index, %	−3.68 (−5.26 to −2.11)	<0.001	−3.10 (−5.88 to −0.32)	0.034
Microvascular obstruction, %	0.26 (0.01 to 0.51)	0.041	0.11 (−0.09 to 0.31)	0.270
Hemorrhage, %	0.28 (0.13 to 0.44)	<0.001	0.15 (0.01 to 0.28)	0.043
Left ventricular ejection fraction, %	−0.72 (−1.38 to −0.07)	0.032	−0.65 (−1.57 to 0.27)	0.168
Multivariable associations,† late CMR (3 months)				
Infarct size, %	0.27 (−0.12 to 0.67)	0.176	−0.36 (−1.10 to 0.38)	0.344
Myocardial salvage index, %	−1.11 (−2.34 to 0.12)	0.079	1.09 (−1.03 to 3.21)	0.318
Left ventricular ejection fraction, %	−0.32 (−0.77 to 0.14)	0.173	0.14 (−0.50 to 0.78)	0.663

Results are additive effects on the CMR parameters. Results at 3 months are adjusted for the initial value of the outcome being analyzed.

* For all baseline CMR outcomes, adjustment was made for the following variables: previous MI, sex, pain‐to‐balloon time, door‐to‐balloon time, thrombectomy, catheterization laboratory pulse pressure, TIMI flow, corrected TIMI frame count, and percent of ST elevation still present after PCI.

† For all follow‐up CMR outcomes, we adjusted for baseline value of the CMR parameter, age, door‐to‐balloon time, glycoprotein IIb/IIIa, TIMI flow, and percent of ST elevation still present after PCI.

### Microvascular Resistance and Left Ventricular Remodeling

Multivariable models with left ventricular end‐diastolic and end‐systolic volumes at 3 months showed no association with IMR after adjustment for initial volumes (Tables 5, 6, and 7).

## Discussion

Our results indicate that after primary PCI for STEMI, microvascular resistance assessed by IMR is a significant predictor of myocardial salvage. Second, microvascular resistance was associated significantly with left ventricular ejection fraction in all patients initially and in patients with anterior MI after 3 months’ follow‐up. Finally, in addition to predicting myocardial salvage, microvascular resistance was a significant predictor of several infarct characteristics, including infarct size, microvascular obstruction, and myocardial hemorrhage. Importantly, IMR provided additive predictive value to established measures of coronary/myocardial reperfusion, including time to reperfusion, coronary flow grades, and electrocardiogram changes. IMR adds early prognostic information at the key time of emergency reperfusion therapy when CMR is not possible, and, potentially, IMR represents a measurable therapeutic target after coronary reperfusion in STEMI.

Because microvascular resistance significantly predicted initial myocardial salvage, we conclude that microvascular patency is a key pathophysiological determinant of the injury/repair response in the infarcted human heart. This observation is a new insight into the pathophysiology of MI and is consistent with previous experimental observations in large animal models.^4^ Independent of other early predictors of myocardial salvage, such as time to reperfusion and initial infarct size, our findings suggest that microvascular function influences the balance between recovery of viable injured tissue and progressive infarction. The pathophysiology of microvascular injury in STEMI is complex and encompasses embolization of proximal thrombus and in situ microvascular thrombosis, as well as extravascular pathologies such as edema and hemorrhage.^27^ We also have demonstrated for the first time that microvascular resistance shortly after coronary reperfusion is associated with subsequent evidence of myocardial hemorrhage as revealed by CMR, consistent with the notion that hemorrhage is an adverse complication of more severe MI.

Our study extends the results of Fearon et al,^10^ who found that IMR predicted left ventricular wall motion score at 3 months after MI in 28 STEMI patients, as well as our own data in a prior study of a separate cohort of patients in whom CMR was used to assess ventricular function.^11^ Our present study confirms these observations in a much larger cohort but also demonstrates that microvascular resistance predicts infarct characteristics, including initial microvascular obstruction and infarct size, especially in patients with anterior MI or an occluded artery.

Intuitively, it makes sense that IMR did not predict final left ventricular ejection fraction in all patients because its predictive value is most likely to be discriminative in patients with large infarctions. For example, a small MI might have little impact on systolic function (eg, occlusion of a left ventricular branch of the right coronary artery) even if microvascular injury is pronounced within distribution of the culprit artery. One other explanation could be that because the sample size is relatively small, there might have been insufficient power to detect a true inverse association between IMR and ejection fraction. We also found that IMR was more closely associated with infarct size than with microvascular injury (ie, microvascular obstruction or myocardial hemorrhage). Because IMR was performed before CMR, we cannot say whether infarct burden is a determinant of microvascular dysfunction or vice versa. However, IMR also correlated with the extent of microvascular obstruction and hemorrhage, which points to the pathological importance of the extent and not just the presence of these abnormalities.^3^

The accuracy and precision of IMR has been established in patients with stable coronary artery disease, but its performance in STEMI has not been fully explored.^9^ We found that IMR was highly repeatable when measured twice 5 minutes apart at the end of primary PCI.

We have shown that IMR is predictive of myocardial hemorrhage as revealed by bright‐blood T_2_‐weighted CMR.^23^ In the current study, IMR was nearly 2‐fold higher in patients with myocardial hemorrhage than in patients without hemorrhage. Myocardial hemorrhage could represent an unintended consequence of evidence‐based antithrombotic and antiplatelet therapies, a manifestation of more severe MI (as suggested by our data), or both. Our other main finding is that early myocardial salvage is a significant predictor of subsequent changes in left ventricular function. This result complements the cohort study by Dall'Armellina et al,^15^ who performed serial CMR scans in 30 STEMI patients over 6 months of follow‐up and found that regression in infarct size during follow‐up predicted the recovery in wall motion. Taken together, both studies indicate that the propensity for improvement in the initial extent of injury (ie, myocardial repair and salvage) is a determinant of future changes in left ventricular systolic function. On the basis of our findings, microvascular patency might permit recovery of salvageable myocardium, whereas microvascular injury might predispose to limited salvage and impaired systolic function in the longer term.

Our results raise the possibility that microvascular injury might be a measurable therapeutic target after coronary reperfusion in STEMI. For this to be the case, IMR should identify patients who might benefit from a specific treatment. Ideally, the treatment should be given at the time of primary PCI, leading to a measurable reduction in IMR or an improvement in prognostically important surrogate measures of microvascular injury.^4–6^ Results from preliminary therapeutic trials during primary PCI for STEMI support the notion that microvascular injury is a therapeutic target.^29,30^

### Limitations

Microvascular patency is only one of several characteristics that influence myocardial salvage and systolic function after MI, and our multivariable models account for only part of this variability, as reflected by the R^2^ values. We could have used a larger signal intensity difference between remote and affected myocardium on LGE CMR to delineate infarct area, but we believe that the main conclusions in our analysis remain valid. Although IMR a multivariable predictor of left ventricular volumes after 3 months, this was not the case after adjustment for volumes measured early after MI. The predictive value of IMR for adverse remodeling needs further prospective study in a larger cohort.

### Conclusions

Microvascular function is a determinant of myocardial salvage in patients with reperfused STEMI and predicts changes in left ventricular function and remodeling. IMR seems to be most informative in patients with an anterior MI or occluded culprit artery.
